# Exploring staff perspectives on caring for isolated hospitalised patients during the COVID-19 pandemic: a qualitative study

**DOI:** 10.1186/s12913-022-09000-3

**Published:** 2023-03-01

**Authors:** Robin Digby, Ingrid Hopper, Leanne Hughes, Doug McCaskie, Michelle Tuck, Kethly Fallon, Peter Hunter, Tracey Bucknall

**Affiliations:** 1 Alfred Health, PO Box 315, Prahran, Melbourne, VIC 3181 Australia; 2 Deakin University, Faculty of Health, School of Nursing and Midwifery, Centre for Quality and Patient Safety Research – Alfred Health Partnership, Melbourne, Victoria Australia

**Keywords:** Patient isolation, Acute hospital, Staff perception, COVID-19, Clinical care, Communication, Person-centred care, Clinical decision-making

## Abstract

**Background:**

Strict isolation of COVID-19 patients to prevent cross infection may inadvertently cause serious adverse outcomes including psychological harm, limitations to care, increased incidence of delirium, deconditioning and reduced quality of life. Previous research exploring the staff perspective of the effect of isolation on patients is limited. The aim of this study is to understand staff perceptions and interpretations of their experiences of the care and treatment of isolated patients and the impact of isolation on patients, families, and staff.

**Method:**

This qualitative, exploratory study is set in a major metropolitan, quaternary hospital in Melbourne, Australia. Data was collected in focus groups with clinical and non-clinical staff and analysed using content analysis. The hospital ethics committee granted approval. Each participant gave informed verbal consent.

**Results:**

Participants included 58 nursing, medical, allied health, and non-clinical staff. Six main themes were identified: 1) *Communication challenges during COVID-19; 2) Impact of isolation on family; 3) Challenges to patients’ health and safety; 4) Impact on staff; 5) Challenging standards of care; 6) Contextual influences: policy, decision-makers and the environment.*

**Conclusion:**

Isolating patients and restricting visitors resulted in good pandemic management, but staff perceived it came at considerable cost to staff and consumers. Innovative communication technology may facilitate improved connection between all parties. Mental health support is needed for patients, families, and staff. Further research using a co-design model with input from patients, families and staff is recommended to determine appropriate interventions to improve care.

Preventing the spread of infection is essential for good pandemic management, but the cost to consumers and staff must be mitigated. Preparation for future pandemics must consider workforce preparedness, adapted models of care and workflow.

**Supplementary Information:**

The online version contains supplementary material available at 10.1186/s12913-022-09000-3.

## Introduction

Response to the COVID-19 pandemic in Melbourne, Australia included a strict lockdown for 4 months in 2020. During this time, visiting in-patients in acute hospitals was severely restricted. A large number of patients presenting with respiratory symptoms or suspected of having the COVID-19 virus were isolated.

Isolation is a gold standard practice in healthcare settings to reduce and prevent the spread of infections particularly those which have the potential to cause serious harm to individuals or the community, or to protect immunocompromised patients from infection [1]. In Victorian acute hospitals, pre-COVID, approximately 12% of patients are in isolation at any one time [2]. A total of 2492 patients were isolated in Victorian hospitals between 25 January and 15 November 2020 [3]. The number of staff employed by the health service increased during this time to meet the increased demands of the pandemic response [4]. A *Patients in Isolation Taskforce* was established by the health service in response to a cluster of falls and other adverse events in isolated patients in early 2020 [4].

Strict isolation of COVID-19 patients to prevent cross infection to other patients and staff may have serious adverse outcomes for patients. Psychological problems, including depression, lethargy, lack of motivation and anxiety, are common [5, 6] and related to uncertainty and loss of control [5, 7], loneliness and reduced social contact [8], and perceived stigmatisation [9]. Patients in isolation have reported poor quality of life [10] and suffer from reduced human contact [7]. However, healthcare professionals who are encouraging and friendly, and engage with patients while attending to tasks, have been shown to make a positive difference [5, 11]. Patients in isolation have a higher risk of developing delirium [7, 12]. Separation from family and familiar environment, inadequate pain management, use of restraints, immobility and sleep disruption contribute to the development of delirium [13].

The physical functioning and rehabilitation of patients in isolation may be neglected [14] leading to deconditioning [15]. Older people can develop sarcopaenia as well as problems directly related to COVID-19 including cardiac, pulmonary, and neurological deficits [16]. Isolated patients potentially receive reduced patient care, delays to care, and less frequent, shorter interactions with healthcare providers [6]. The prevalence of malnutrition in hospitalised patients with COVID-19 is high [16]. Adverse events, including falls, pressure injuries, medication errors and fluid imbalance are common [8, 10].

Hospital staff caring for patients during a pandemic are at increased risk of adverse mental health outcomes [17–19]. Additional stressors in an already high-pressured clinical environment include potential exposure to COVID-19 and risk to their families, anxiety about the future [20], overwhelming workload [21], challenges to the standard of care [22], and moral distress [23].

In Australia, previous experience with pandemic management is limited. To optimally manage future similar situations, more information is required on the effect of isolation on key stakeholders. Previous studies have explored this topic from the viewpoint of patients demonstrated in a systematic review and meta-analysis [10] which revealed that patients in isolation rooms commonly felt distressed, alienated, a burden to healthcare staff and uninformed about infection prevention. Additionally, patients in isolation experienced fewer bedside visits and there could be limitations to care [10]. There is very limited research about the effect of isolation on patients and families from the perspective of staff who deliver and are accountable for patient care [24, 25]. Staff working closely with this patient group can provide unique insight into the issues connected with isolation and inform potential solutions. This study elicited the opinions of clinical and non-clinical staff working with patients in isolation.

## Methods

### Aim

The aim of this study is to understand staff perceptions and interpretation of their experiences of the care and treatment of isolated patients and the impact of isolation on patients, families, and staff.

### Design

This qualitative descriptive study collected data from focus groups with clinical and non-clinical support staff, and is reported using the Consolidated Criteria for Reporting Qualitative Research (COREQ) [26].

### Setting

The setting is a 600-bed major metropolitan, tertiary referral and teaching hospital in metropolitan Melbourne which played a major role in the management of the COVID-19 pandemic. The study was focused on the Melbourne lockdowns between 31st March 2020 and 27th October 2020.

### Participants

A purposive sample of participants from nursing, medicine, and allied health (AH) including physiotherapists, occupational therapists, dietitians and speech pathologists, and non-clinical support services including food services, porters and environmental staff was recruited through advertising in staff newsletters, via email distribution and with the assistance of managers. All staff including non-clinical staff, working in direct contact with patients in isolation were eligible to participate.

### Instruments

Eight focus groups, including three groups of nurses, two groups of doctors, two allied health (AH) groups, and one group of support services staff, all including staff with a range of roles and seniority, were conducted 8th October- 2nd November 2020 via videoconferencing by clinicians in the field who were known to some of the participants. One researcher, a female RN (XX) with previous interviewing experience participated across all focus groups. Mutual respect was discussed at the commencement of each session, and the participants were invited to contribute their views either in the discussion or via the chat box. An aide-memoire focused questions on ascertaining the effect that isolation and the severe limitation of access had on patients, families, and staff (Table 1). The participants were invited to discuss any patients in isolation, not only those with COVID-19. Duration of focus groups was 45–60 minutes.

**Table 1:** Aide-memoire

1. Can you describe your most recent experience while caring for a patient in isolation? 2. Can you describe what worked well? • Care Processes such as ward rounds/ staffing levels/ staffing roles/PPE • Environment • Clinical outcomes • Team relationships/communication • Patient experience 3. Can you describe the challenges? • Care Processes such as ward rounds/ staffing levels/ staffing roles/PPE • Environment • Clinical outcomes • Team relationships/communication • Patient experience 4. How did the changes to the ward environment affect your ability to do your work? • isolation rooms • Creation of a COVID rooms with donning and doffing stations • PPE monitors • What was the most challenging aspect overall? • What was the most satisfying? • In hindsight, if something could be done differently what would you recommend? 5. Can you provide any suggestions as to how to overcome some of the challenges you mentioned when caring for the patient in isolation? 6. Is there anything else that you would like to add that has not been discussed?	

### Data analysis

Focus groups were audio-recorded and transcribed verbatim. Members of the research team analysed several transcripts each and discussed the themes and sub-themes from independent analysis as a group. One researcher (XX) conducted further analysis of all focus groups using qualitative content analysis [27] and NVivo software for data management [28]. The themes and subthemes from the research team and researcher XX were combined and discussed with the group. Refinements were made until there was consensus.

### Findings

A total of 58 staff were interviewed including 24 nurses, 16 allied health clinicians (5 from nutrition and dietetics, 3 occupational therapists, 4 physiotherapists, 2 allied health assistants (certificate level staff who support allied health professionals with administrative tasks and overall care), 1 social worker and 1 department director), 9 medical doctors and 9 non-clinical support staff. The support staff included food services assistants, environmental staff and porters.

Six main themes were identified: *1) Challenges to patients’ health and safety; 2) Challenges to standards of care; 3) Impact of isolation on family; 4) Impact on staff; 5) Contextual influences: 157 policy, decision-makers and the environment; 6) Communication challenges during COVID-19* (Table 2).

**Table 2:** Themes and sub-themes

Challenges to patients’ health and safety	Challenges to standards of care	Impact of isolation on family	Impact on staff	Contextual influences: policy, decision-makers, and the environment	Communication Challenges during COVID-19
Patient challenges	Patient care	Impact on family	Exposure to the virus	Organisational decision-making	Organisational communication
Discharge safety	Palliative care	Educating the carers	Workload	Infrastructure	Communicating with families
Patient nutrition	MET calls		Support and Teamwork	Visiting rules	Communicating with patients
Patient mobility	Patient outcomes		Personal Protective Equipment		Communication between staff
					Communication technology

### Theme 1: challenges to patients’ health and safety

#### Managing the impact of social isolation on patients

Patients in isolation were deprived of the usual family support, seriously affecting some patients who had previously depended on family for care at home, were cognitively impaired or from non-English-speaking background. ‘*The loneliness and the frustration… The confusion would be increased…’*(Nurse). Younger patients who were confident to use electronic devices coped better. ‘…*we had a lot of tech savvy young people on the ward who never needed us to assist them in communication. They would Skype or WhatsApp their family and have them there for the ward round…’*(Doctor). The impact of isolation on patients was noted by the staff: *‘the mental side of it was often much more of a barrier to their recovery than the actual physical illness itself… the prolonged isolation, not being allowed to have your door open… it was quite hard to find ways to stimulate those patients and to encourage them.* (Nurse).

#### Barriers to patient nutrition

Food services staff would leave meal trays outside patient rooms ready for clinicians to take in, however this often didn’t occur until much later when the food was cold. *‘There’s been so many times where you go past the patient’s room, and it’ll be 2:00 PM and their lunch from hours ago is just sitting outside….’* (Doctor). Additionally, many patients received generic default meals because they were unable to complete their meal orders. Families often play an important part in encouraging patients to eat. Patients come from diverse cultures and in normal circumstances, families can bring food for the patient which is more culturally appropriate. *‘… the inability for family members to bring in food…showed just how much we do rely on those external family members and friends to provide culturally suitable food for our patients*.’(AH).

#### Constraining patient mobility in isolation

Confining patients to their rooms limited mobility. Patient endurance and fitness suffered. ‘*Patients were very sedentary, often weren’t sat in the chair for meals or prompted to be active at all’* (AH). The requirement to don PPE before entry to a patient room caused delays when responding to patient falls *‘If the patients are high falls risk or other issues where you have to run in quickly that’s another stress to add onto it, because you’re trying to get in there quickly, but you’re also trying to do your PPE properly’.*(Nurse). Staff wanted to be there for patients who needed them, but it could be difficult *‘just getting into the room in time was not always possible, and that was quite hard for us too’* (Nurse). Physiotherapy was limited because patients remained in their rooms ‘…*not being able to take a patient outside of their room, not being able to assess function more than 10 metres in their room. Not being able to do stairs assessments’* (AH).

### Theme 2: challenges to standards of care

#### Curtailing direct patient care

Telehealth was utilised to deliver some forms of care. Transdisciplinary practice was introduced to reduce the number of staff visiting patients. In some instances, minimising staff access to patients affected the standard of care. Staff perceived that sometimes patients received less attention than they required ‘*It sounds terrible, but because you’re only going in to do the essentials because you want to limit your time … they only really see you when they need something’* (Nurse).

Participants reported that isolation could potentially lead to sub-optimal or delayed treatment, and patient deconditioning. *Putting on and taking off PPE is really time consuming and sometimes it makes it difficult to respond to patients needs quickly* (Nurse). In some cases, the effect of social isolation contributed to patient decline. *No (family) was able to come in, and the patient was not really eating much at all… he just kept saying was, “I want my wife, I need my wife”* (AH). Patients with impaired cognition or mental health disorders were sometimes restrained to keep them isolated. ‘*Patients received pharmacotherapy and were physically restrained, who would never have needed either of those interventions had they not had COVID …that’s not the standard of care that I want to deliver’* (Doctor). To limit exposure of equipment and furniture, isolation rooms tended to be spartan ‘*I walked into that room and honestly, it looked like the building was being vacated, … I felt we weren’t even meeting her basic human needs’* (Doctor). Patient isolation was accepted as necessary, but difficult. *‘The idea that you were actively making a patient’s health worse for the sake of the broader community is not a very satisfying one*’ (Doctor).

#### Restrictions on palliative care

Visiting to patients receiving end-of-life care was extremely limited, especially for those who were COVID positive. This was difficult for everyone involved and did not align with the person-centred focus of good palliative care. Staff reported feeling distressed witnessing the trauma to patients and families. *‘(The mother) was adamantly refusing to leave the hospital. There was a conversation about whether or not we will be getting security to remove her from the room. It was just a horrendous experience … normally palliative care is the one medical specialty where everything you do is supposed to make the patient’s life better* …’ (Doctor).

Staff reported that families were having to choose who went in to see a dying relative. ‘*We made them choose which family members were allowed to come… and I found that very uncomfortable to make a family where the patient has six children and only allow three of them to visit their dying parent’* (Doctor). Another case was reported of a family who initially wanted active care but chose end-of-life care for their relative so that they could come into the hospital. ‘*They only said that they were happy for end-of-life care because they wanted to come into the hospital… and I’m just not sure that’s a good enough reason for a family to decide on palliative care’* (Doctor).

#### Challenges with medical emergency team (MET) calls

The number of staff entering the room during MET calls was kept to a minimum and the resuscitation trolley kept outside the patient room. Having to rely on staff outside the room for drugs and equipment created delays. *‘…when staffing is short on the night shift and you’re in a complicated MET call you’re relying on people outside the room to grab things that you need in a timely manner’* (Nurse).

### Theme 3: impact of isolation on family

#### Minimising the impact of isolation on family

Participants reported that isolating patients was extremely distressing for the families, patients, and staff. Many family members struggled to cope with separation from very unwell relatives. Limiting or preventing family access to a dying patient was especially difficult. The important role that family plays in patient care was starkly evident. ‘*… we know the importance of having their loved ones there… we need to as an organisation prioritise having these family members come in and come up with a strategy’* (Doctor).

#### Educating the carers remotely for patient discharge

During the lockdown, allied health home visits were not permitted creating problems assessing the patient’s home environment and the capability of the patient and carer to manage after discharge. Carer education was generally conducted remotely but it was difficult to have confidence in discharge safety. *‘… people were trying to do carer training via Telehealth which was very challenging, not only to establish someone’s competence and safety in those tasks, but also emotionally for the families’* (AH).

### Theme 4: impact on staff

#### Fear of exposure to the virus

The pandemic impacted on both staff work and homelife and caused considerable stress for many. Uncertainty about the nature of the virus and the risk to themselves and their families, increased workload, isolation from family and friends, and adapting to new ways of working all contributed to anxiety. ‘*We are living in constant fear of exposure to the virus … you’re always aware of the danger that you could pass it to your loved ones’* (Nurse). Staff were sometimes absent from work because of the need to be tested which created rostering challenges. *‘A lot of people wanted to get tested because they’re worried about their families, which meant staffing difficulties because they wouldn’t be able attend to work before the test result is given negative …*’ (Support services).

#### Managing the extra workload

Isolating so many patients created workload challenges. “*… extra thinking and problem-solving – that probably took more of an emotional drain... I would be absolutely exhausted from having so many conversations around different patients and finding creative ways in how to work with them*” (AH). Extra time was required to don and doff PPE, alternative strategies were needed for formerly straightforward procedures and additional time was spent communicating with families. In specific areas, staffing numbers were boosted to help with the increased workload and staff work patterns were adjusted. Support services instigated a more rigorous cleaning schedule to comply with pandemic requirements, adding to their workload. ‘*With the isolation cleaning and the high touch point cleaning that we’re doing … super cleaned and isolated wards are all marked prior to the super cleans and then checked..*. (Support services).

#### Staff support and teamwork

Staff reported frequently feeling distressed witnessing the anguish of patients and families *‘She arrived and the patient died about 2 minutes prior to that … they were very appreciative of my caring and my communication. But that didn’t help me feel any better’* (Doctor). Not allowing family to be with a dying relative was always difficult. ‘*It’s a really sad experience… the patient’s alone dying and their next of kin can’t visit them … it was really challenging* (Nurse).

Participants found support from different avenues including colleagues and external businesses who donated meals or gifts for staff. The organisation sent emails of encouragement and gratitude, but the most effective support came from direct managers and colleagues. ‘… *our nurse unit manager has been really good at just talking to everyone, keeping everyone informed, checking in….*’ (Nurse). Teamwork improved, and participants reported an increased camaraderie as they faced unfamiliar, difficult situations together. ‘…*where we’ve actually had end of life care on the wards and being able to come together as a team … to acknowledge how difficult certain patients’ deaths have been’* (AH).

Ward-based allied health and medical teams were formed. Some allied health staff adopted transdisciplinary practice facilitated by telehealth to reduce staff movement and exposure to the virus. ‘*We’re no longer siloed into OT, PT nutrition, speechies. We are beginning to share our skills more and do joint assessments and management’* (AH). Support services rapidly recruited and trained extra staff to cope with the increased demand. ‘We had a number of displaced staff from the hospitality industries that we were able to recruit and train’ (Support Services).

Some staff received support from psychologists made available for this purpose: *‘Then into the second wave, I think everyone really struggled and found it a lot harder … so we actually had a catch up with the psychologist at [hospital], which some people found beneficial just to voice what they were feeling and what their concerns were* (Nurse).

#### Managing the requirements of PPE

Wearing PPE could be hot and uncomfortable, and cause pressure injuries on noses, ears, or cheeks, ‘*Staying in PPE for long periods is uncomfortable…. It gives you pressure areas from the masks, and the gowns can become pretty sweaty...’* (Nurse). The requirement to wear PPE added extra time to tasks and triggered concern about the risk of contracting the virus if mistakes were made. ‘*You have to protect yourself and take time to apply all the PPE and not rush everything. Because there’s always fear of catching the virus’* (Nurse). The management of discarded PPE in infectious waste bins was a logistical challenge for support staff ‘*…some of the challenging things - the amount of waste infectious waste that was being generated*… (Support Services).

### Theme 5: communication challenges during COVID-19

#### Impacts of a dynamic situation on organisational communication

Emerging knowledge of the virus and its transmission required the organisation to continuously revise and update protocols. Hospital executive provided email bulletins and staff updates via videoconferencing. The hospital complied strictly with the Chief Health Officer’s directives, however the rapid pace of change sometimes led to a lag in information dissemination. ‘…*there was a disconnect between what was said on the hotline and website to what was actually protocol’* (Doctor). Staff reported interpreting directives differently and perceived that the guidelines changed from shift to shift.

#### Remote communication with families

Communication with families was prioritised to promote information-sharing and engagement. *‘I don’t necessarily need to interact with those patients face to face, but more to support the families who are normally very anxious …’*(AH). Regular phone calls or videoconferencing between clinicians and families was reasonably effective, although it added to staff workload and had limitations. ‘*I’ve got a 23 inpatient list and it will take me 2 hours to call the family members’* (Doctor). Telehealth took extra time to organise *‘They do try and do Telehealth. It’s nice, but it’s time consuming for nursing staff to set up’* (AH). Managing the technology for videoconferencing was beyond some family members. ‘*…the people on the other end outside of the hospital may have limited technical skills or may not have the right devices … (*Nurse).

#### Hindering communication with patients

The physical barrier created by PPE hid clinicians’ facial expressions and individuality which hindered communication. ‘*Patients who are delirious or have cognitive deficits, we’re all wearing masks, everybody looks the same’* (AH). Clinicians were urged to minimise time spent in patient rooms to reduce their exposure to infection, limiting the time available to engage with patients. Building rapport was especially challenging with patients who had cognitive, vision or hearing impairment.

##### Communicating key messages to staff

Videoconferencing and short updates at shift handover enhanced staff communication. Minimising staff movement within the hospital meant that home team allied health and medical staff were more available on the wards. Staff were kept updated about the rapidly changing regulations and protocols using technology. ‘*What’s been really beneficial is the use of [videoconferencing] for journey boards …we had the nurse in charge and the allied health team lead and then all the members of allied health log on.*’ … (AH).

##### Employing new communication technology

Technology improved communication. ‘… *it pushed everyone into [videoconferencing] and using technology in a whole new way … we’ve fast forwarded 10 years in terms of how connected we are…*’ (AH). Staff used videoconferencing for meetings, staff updates and communication between clinicians. Ward rounds were conducted with one or two staff visiting the patient with a videoconferencing device, and the remaining team members connecting remotely. ‘*The reg [istrar] or I would go into the room with the iPad and then everyone is on the round, the pharmacist is in the office, the intern is in a separate office’* (Doctor). However, not being able to conduct aspects of physical examination such as listening to a patient’s chest or examining a wound was a shortcoming, which affected the education and experience of junior medical staff. *‘The consultant asked me to examine the patient for him and I had a bit of an embarrassing, flustered experience where I hadn’t done it for so many weeks … I was a bit out of practice’* (Doctor). It was especially difficult for some allied health professions who would normally treat patients in different areas of the hospital but were restricted to reduce transmission risk. Photos and videoconferencing were not always an adequate substitute *‘… I’m relying on photographs, FaceTime… at the end of the day when you’re trying to fix a feeding tube, it’s not really conducive…* (AH).

### Theme 6: contextual influences on policy, decision makers and the environment

#### Making decisions during unchartered times

The Department of Health, hospital executive and the physical environment all influenced the management of patients, families, and staff during the pandemic. It was difficult for those in authority to navigate this unfamiliar territory. Decisions were made quickly to address issues as they arose. ‘*Sometimes it seemed like the legislation around who could come and when was very arbitrary … it doesn’t really seem to be aligned with clinical risk’* (Doctor).

#### Infrastructure barriers to caring for isolated patients

Hospital buildings were not designed to cater for so many isolated patients and had some limitations including opaque doors which made it impossible for patients to be observed from outside the room. ‘*Rooms that had this frosted glass for patient privacy, it was a huge barrier because you weren’t allowed to open the room and cast a glance to see if the patient looked ok’* (Doctor). Shared bathrooms required full cleaning after each use leading to delays to personal care.

The dramatic increase in the use of PPE created an enormous amount of hazardous waste and caused a logistical problem for support services ‘*A limited confined space for all for the waste to be dispensed from the dock to the trucks …we get this surge of clinical waste coming through* …’ (Support Services).

#### Restricted visiting

Some participants considered the blanket rule governing visitors to be too harsh and lacking compassion. There was little leeway for staff to manage difficult individual circumstances despite witnessing the trauma the restrictions caused. There were different visitor restrictions for COVID positive, COVID at risk, and non-COVID patients. ‘*On the website it was clearly stating that there could be up to two visitors a day … but then nurse in charge and clinical service director would have a different opinion’* (Doctor).

## Discussion

This study provides insight from clinical and non-clinical staff into caring for patients in isolation. A strength of the study was the diversity of experience shared by participants who were from different clinical disciplines and non-clinical roles. Support services staff were focused on the logistics of providing cleaning and meal services to patients in isolation and removing waste. Clinical staff – nurses, allied health and doctors – were more closely involved with patients’ biopsychosocial needs which was reflected in their views. However, all disciplines were aware of the psychological and practical effects of isolation on patients. Compliance with pandemic restrictions created considerable challenges to ensure that patients received the best possible care. Negative impacts reported included decline in standard of care, distress for families unable to see their loved ones, challenging work environments and moral distress for staff. Staff health and well-being suffered. Some positive impacts included use of new technology, creative problem solving, staff teamwork and camaraderie, and regular communication with families. Staff worked hard to alleviate the negative consequences of the restrictions on the people involved.

Maintaining the standard of patient care during isolation was a pre-existing problem [7, 8, 10, 29] recognised by the hospital by implementing a task force. The experience of mass isolation during the pandemic accelerated the need to identify creative solutions to be applied to isolation in non-COVID times. Like healthcare organisations globally, innovative use of technology changed communication modes between healthcare professionals, with patients and families and the wider community [30, 31]. Adapting methods to deliver effective communication that was suitable for the new conditions was a priority. Hospital leaders used a combination of electronic briefings, short emails and limited in-person contact to communicate regular updates to clinicians, an approach also used successfully in the UK [32] and elsewhere [33]. Previous research identified that uncertainty about the course of the pandemic, frequent changes to processes and conflicting information, added to staff stress [17, 34]. Considerable skill is required to have difficult conversations with patients and families via telehealth or on the phone; a skill not shared by all clinicians [31].

Personal protective equipment hinders communication between clinicians and patients, especially masks, which obscure the view of the speaker’s mouth and facial expression and reduced voice volume [35]. PPE not only decreases the wearer’s auditory perception and affects the ability to make contact with others [29], but reduces situational awareness which intensifies with an increase in PPE discomfort [35]. Face shields and goggles introduce glare which further limits the view of the speaker’s face [36] and creates a communication obstacle between team members and with patients [37]. The use of personal protective equipment can be depersonalising and alarming to patients, especially those with underlying cognitive impairment [38]. Previous research has reported similar issues to those found in our study including delays in responding to MET calls [39, 40], delivering meals to patients, and preventing falls because of the need to don PPE before entering an isolation room [5].

Decreased staff interactions and family presence meant some patients became lonely and anxious. Marler and Ditton [41] reported that patients who don’t recognise their attendants may develop increased loneliness, confusion and anxiety leading to a liminal experience. A close therapeutic relationship with nurses and other clinicians can mitigate feelings of being an outsider, of being marginalised and kept separate from others [42] but closeness is difficult to foster under these circumstances [34]. Staff were also impacted by the decreased interactions with patients and families.

Telehealth or phone contact only partly alleviated the distress and staff reported some patients felt abandoned. Voo, Senguttuvan [43] suggested that more energy should be devoted to supporting isolated patients with family presence - physical, virtual, or surrogate - although this can create a further burden on an already over-extended workforce. The effect of the separation of dying patients from their families was perceived as particularly traumatic and not easily substituted with telehealth. The experience could potentially cause on-going psychological pain and posttraumatic stress to families [44] and to staff [45].

Denying families access to their relatives who were critically unwell or had other comorbidities such as dementia, sensory impairment or were non-English speaking caused significant moral distress to staff. Moral injury results from actions or lack of them which violate one’s moral or ethical code [23, 46]. The ethical principles of clinical care during a pandemic are complex. The protocols must be followed; however, clinicians may be conflicted when institutional restrictions prevent them from caring the way they consider best for individual patients [47]. Extreme examples of moral injury in which a person feels that their core values are eroded can lead to posttraumatic stress disorder (PTSD) although intervention can sometimes be preventative by cultivating resilience [23]. The need for the staff to debrief about their moral distress during this study highlights the importance of formal and informal debriefing after a major event.

Staff reported an increased camaraderie as they faced difficult situations together. In many cases, workload and stress increased, however teamwork and support from colleagues and managers made the situation more bearable. Informal peer support can alleviate the need for formal intervention, prevent burnout [33], and reduce the stigma of stress [21]. Reinforcing social bonds between colleagues and supervisors is protective of mental health [20], and can facilitate help seeking when appropriate [21]. Feeling protected and supported has been found to promote role confidence and self-efficacy in frontline staff [33, 34].

The COVID-19 pandemic has presented hospitals with an unprecedented challenge, organisationally and individually [17, 48, 49]. However, the experience has identified areas of strength and others that require improvement. Isolating large numbers of patients necessitates alternative models of care and different workflow practices, some potentially enabled by technology, to ensure that a high standard of care is maintained [50]. Patient and family-centred care principles must be embedded into care for all patients, including those in isolation [11]. A more robust system for virtual visiting, and more flexibility with in-person visiting restrictions is recommended [51]. Workable solutions to these problems could be sought using co-design methodology [52], with input from patients, families and staff. The restrictions both in the hospital and the community has had a significant impact on staff health and well-being. Staff were especially vulnerable to emotional distress, given their risk of exposure to the virus before vaccination was established. Appropriate support must be provided to mitigate against long-term psychological effects and maintain a robust workforce [53, 54].

### Strengths and limitations

A major strength of this study was the large, interdisciplinary clinical and non-clinical staff participation in focus groups, shortly after a city lockdown had ceased. Limitations include the recruitment of participants from a single metropolitan hospital, at a snapshot in time, hence the findings and implications may be limited to similar settings.

## Conclusion

Although isolating numerous patients and severely restricting visitors resulted in good pandemic management, health care workers perceived considerable impact on patients, families, and staff. The insight staff provided will inform planning for future pandemics. Communication technologies and strategies, mental health support for patients, families and staff, and adaptable infrastructure are pre-requisites. Further research is required to understand what improvements are needed from the patient and family perspective and to determine the effectiveness of interventions on isolated patient care.

## Supplementary Information

**Additional file 1.**

## Data Availability

The datasets used and analysed during the current study are available from the corresponding author on reasonable request.
